# Effects on Photosynthetic Response and Biomass Productivity of *Acacia longifolia* ssp. *longifolia* Under Elevated CO_2_ and Water-Limited Regimes

**DOI:** 10.3389/fpls.2022.817730

**Published:** 2022-03-31

**Authors:** Muhammad Mansoor Javaid, Xiukang Wang, Singarayer K. Florentine, Muhammad Ashraf, Athar Mahmood, Feng-Min Li, Sajid Fiaz

**Affiliations:** ^1^Department of Agronomy, College of Agriculture, University of Sargodha, Sargodha, Pakistan; ^2^College of Life Sciences, Yan’an University, Yan’an, China; ^3^Future Regions Research Centre, Federation University Australia, Mount Helen, VIC, Australia; ^4^Institute of Molecular Biology and Biotechnology, The University of Lahore, Lahore, Pakistan; ^5^Department of Agronomy, University of Agriculture Faisalabad, Faisalabad, Pakistan; ^6^State Key Laboratory of Grassland Agroecosystems, School of Life Sciences, Institute of Arid Agroecology, Lanzhou University, Lanzhou, China; ^7^Department of Plant Breeding and Genetics, The University of Haripur, Haripur, Pakistan

**Keywords:** elevated CO_2_, photosynthetic processes, water use efficiency, photosystem II, drought, *Acacia longifolia* ssp. *longifolia*

## Abstract

It is known that the impact of elevated CO_2_ (eCO_2_) will cause differential photosynthetic responses in plants, resulting in varying magnitudes of growth and productivity of competing species. Because of the aggressive invasive nature of *Acacia longifolia* ssp. *longifolia*, this study is designed to investigate the effect of eCO_2_ on gas exchange parameters, water use efficiency, photosystem II (PSII) activities, and growth of this species. Plants of *A. longifolia* ssp. *longifolia* were grown at 400 ppm (ambient) and 700 ppm (elevated) CO_2_ under 100 and 60% field capacity. Leaf gas exchange parameters, water use efficiency, intrinsic water use efficiency, instantaneous carboxylation efficiency, and PSII activity were measured for 10 days at 2-day intervals. eCO_2_ mitigated the adverse effects of drought conditions on the aforementioned parameters compared to that grown under ambient CO_2_ (aCO_2_) conditions. *A. longifolia*, grown under drought conditions and re-watered at day 8, indicated a partial recovery in most of the parameters measured, suggesting that the recovery of this species under eCO_2_ will be higher than that with aCO_2_ concentration. This gave an increase in water use efficiency, which is one of the reasons for the observed enhanced growth of *A. longifolia* under drought stress. Thus, eCO_2_ will allow to adopt this species in the new environment, even under severe climatic conditions, and foreshadow its likelihood of invasion into new areas.

## Introduction

A substantial increase in atmospheric CO_2_ level has already occurred since the Industrial Revolution, and it is predicted that it will approach 700 ppm by the year 2050 (IPCC, 2018). This increase in atmospheric CO_2_ will cause global warming (Boucher et al., 2009) that leads to water scarcity, a key constraint of crop productivity. Despite that, elevated CO_2_ (eCO_2_) is believed to increase the photosynthetic rate and suppress their photorespiration (Li et al., 2019). eCO_2_ increased the photosynthesis and stimulated biomass and yield parameters (Kirschbaum, 2011) along with decreased water use per unit area of vegetation by partial stomatal closure (Pastore et al., 2020). Studies have also shown that eCO_2_ causes a 20–30% increase in net assimilation rate, together with an increase in dry matter and yield of crops (Cruz et al., 2018; Pan et al., 2018; Xie et al., 2018). Increasing atmospheric CO_2_ has significant effects on physiological processes related to plant growth, even though responses will be modified under drought stress (Qaderi et al., 2006, 2013). It is well documented that enhanced biomass production and alteration in photosynthetic activity and water relation under eCO_2_ are dependent on the availability of other limiting resources (Robredo et al., 2007). According to Elgawad et al. (2015), eCO_2_ has been shown to mitigate the stress impact of drought, and they quote examples of how the reduction of drought stress affects barley and alfalfa crops. Despite these beneficial effects on crops, ongoing increases in atmospheric CO_2_ may have important consequences for weed-crop competition, leading to subsequent yield losses (Ziska et al., 2019).

It has been noted that climate change further increases the risk of plant invasiveness by facilitating a change in the ecosystem through the increased competitiveness of the weed under eCO_2_ conditions (Bradley et al., 2010; Le et al., 2019). Previous studies have shown that invasive plants exhibit greater climatic change tolerance in invaded habitats compared to native species due to evolutionary plasticity (Clements and Ditommaso, 2011; Davidson et al., 2011). In this respect, eCO_2_ and water stress, sowing to climate change, can directly affect plant physiology and morphology (Lobell and Gourdji, 2012; Salazar-Parra et al., 2012). It is postulated that because invasive species benefit from eCO_2_, this will enable them to acclimatize to their new environments in the future (Dukes et al., 2011; Pastore et al., 2020). It is predicted that rising atmospheric CO_2_ may cause global warming, which could be the basis of change in precipitation patterns and drought occurrence, and could possibly affect those regions not currently subjected to drought (Robredo et al., 2007; Dai, 2013; Dai et al., 2018). It is also postulated that different plant species showed different responses with rising atmospheric CO_2_ concentration under drought conditions (Pastore et al., 2020). Researchers have observed that plants are grown under eCO_2_ and dry more slowly as water is withheld due to lower stomatal conductance and transpiration rate (Dikšaitytė et al., 2019; Pastore et al., 2020).

*Acacia* species, which are native to Australia, have been deliberately exported for some years to a number of temperate countries in order to stabilize degraded soil (especially sand dunes) for ornamental purposes and fuelwood (Kull et al., 2011). However, the aggressive growth properties of most of the *Acacia* species have made them particularly invasive (Corlett, 2010), with the result that these species are currently included in the Global Invasive Species Database. It is well established that invasive alien species affected the growth and wellbeing of native species by altering the ecosystem and introducing direct competition for resources (Werner et al., 2008; Alba et al., 2019). According to Blossey and Notzold (1995) hypothesis, competitive abilities can be maximized by increasing vegetative growth or reproductive growth for success in a particularly new environment. This alteration in the mix of biota presents environmental managers with an urgent challenge to stem the infiltration of invasive species in order to maintain indigenous biodiversity (Qaderi et al., 2013). In many situations, it is evident that these invasive plant species are considered to be the major cause of biodiversity loss and are also thought to contribute to global environmental change (Bellard et al., 2014).

*Acacia longifolia* ssp. *longifolia*, which is the focus of this article, belongs to the *Fabaceae* family and is commonly known as either Sydney golden wattle, long-leaved wattle, or shallow wattle (Marchante et al., 2010). It is indigenous to South Eastern Australia (Costermans, 1981), but currently it is widely distributed throughout Australia (Marchante et al., 2015). Whilst sandy coastal areas are the preferred habitat of *A. longifolia*, it also grows successfully in woodlands, grasslands, and along roadsides (Marchante et al., 2010). Its ability to fix nitrogen enables this species to colonize successfully in nutrient-poor soil (Sampaio, 2019). Due to its aggressive growth habit, this species is currently recognized as a problematic invasive plant in adjoining native areas in Australia, as well as in introduced environments in Portugal, Southern Africa, and Spain (Souza-Alonso et al., 2018). Many reports have indicated that this species can outcompete native plants by rapid growth even under disturbed conditions (Osunkoya et al., 2005; Marchante et al., 2015). This invasive character of *A. longifolia* ssp. *longifolia* can be attributed to its prolific annual seed production and physical seed dormancy due to its hard seed coat, which facilitates its soil seed bank persistence and its consequent emergence in times of stress (Welgama et al., 2019).

The interest of this article is that many species of *Acacia* respond to eCO_2_ concentrations and record increased growth as a result of enhanced carbon assimilation (Evans et al., 2000; Le et al., 2019). In this respect, Evans et al. (2000) studied the effect of eCO_2_ on photosynthetic activity of 10 *Acacia* species (i.e., *Acacia aneura*, *Acacia colei*, *Acacia coriacea*, *Acacia tetragonophylla*, *Acacia irrorata*, *Acacia dealbata*, *Acacia mearnsii*, *Acacia implexa*, *Acacia melanoxylon*, and *Acacia saligna*) under normal irrigation conditions. Except for *A. aneura*, results revealed that growth and gas exchange measurements of all species were enhanced under 700 ppm CO_2_ compared to that under 350 ppm, with the degree of enhancement being species-specific. *A. longifolia* ssp. *longifolia* was not included in the previous study, but other studies have revealed a wealth of information on its ecology and biology. Nonetheless, more information about physiological processes and biomass production regarding *A. longifolia* ssp. *longifolia* will increase the understanding of the potential future impacts of climate change on this species. As a consequence, this study was designed to investigate the effects of drought on *A. longifolia* ssp. *longifolia* under two atmospheric CO_2_ concentrations (400 and 700 ppm), noting changes in physiological processes, water use efficiency, and growth.

## Materials and Methods

### Experimental Site and Sowing Conditions

Experiments were conducted at Federation University, Mt Helen, Australia (37°37.39°S, 143°53.27°E) in two CO_2_ chambers (2.1 m length, 2.1 m width, and 2.0 m height) (Steridium Pty Ltd., Brendale, QLD, Australia). One CO_2_ chamber was set at 400 ppm CO_2_ concentration [ambient CO_2_ (aCO_2_)], whilst the other was set at 700 ppm CO_2_ concentration (eCO_2_). The average chamber temperature was maintained at 22°C day/18°C night alternating temperature with 60% humidity. Mature seeds of *A. longifolia* ssp. *longifolia* were collected from the Grampians National Park Victoria (37°12.16°S, 142°23.35°E). Two moisture levels (well-watered and drought) were also maintained in each CO_2_ chamber. Twenty plastic pots (13 cm wide and 14 cm height) were filled each with 800 g of 2:1 mixture of garden soil and commercially available potting mixture. The seeds of *A. longifolia* ssp. *longifolia* were surface-scarified with sandpaper No. 1000 to remove the physical dormancy, imposed by the seed coat. Five scarified seeds were sown in each pot.

### Effects on Photosynthetic Response by Drought Conditions and Elevated CO_2_ Levels

Out of 20 pots, 10 pots were placed in the chamber with aCO_2_ concentration, and the remaining half were placed in the chamber with eCO_2_ concentration. The pots were placed in large plastic trays, and water was added to these trays to reduce the disturbance of the potting mixture until emergence occurred. At the four-leaf stage, the seedlings were thinned to two per pot. After 30 days from sowing, the first measurements of gas exchange parameters were recorded, and these measurements were termed “zero-day.” Subsequently, moisture treatments of well-watered and drought were commenced. The well-watered treatments were maintained at 100% field capacity and drought treatments at 60% field capacity. These water regimes were selected as different plant species showed variable responses with eCO_2_ concentration under drought conditions. The water holding capacity of the soil used in the pots was calculated according to the method suggested by Bajwa et al. (2017). Of the 10 pots in each chamber, half were subjected to well-watered, and the remaining half were subjected to drought. The pots were weighed in order to maintain the field capacity levels, and the weight of the growing plant was much smaller than that of the soil in pot. There were five replications (one pot for each replication) for each treatment, and each replication consisted of two plants. In the drought treatments, water was withheld until day 8 in both CO_2_ chambers, after which drought treatment pots were re-watered to investigate the recovery response of the *A. longifolia* ssp. *longifolia* plants. The amount of water added was calculated based on pot weight, and 60% field capacity was maintained till day 10. In the well-watered treatments, water was added on alternate days. To evaluate the effect of CO_2_ and drought, physiological parameters were measured using the LI-COR portable infrared CO_2_ gas analyzer (LI-6400 XT portable photosynthesis system, LI-COR, Biosciences, Lincoln, Nebraska, United States). Measurements were recorded with the following adjustments of LI-COR: block temperature was set at 20°C, photosynthetic photon flux density (PPFD) was 1,000 μmol m^–2^ s^–1^, leaf cuvette area was set at 2 cm^2^, and the flow rate was adjusted at 500 μmol m^–2^ s^–1^. The CO_2_ concentration in the chamber was noted before each measurement. Net photosynthetic rate, stomatal conductance, transpiration rate, and intercellular CO_2_ concentration were measured on alternate days. Water use efficiency was calculated by dividing the net photosynthetic rate with transpiration rate, intrinsic water use efficiency was calculated by dividing the net photosynthetic rate with stomatal conductance, and instantaneous carboxylation efficiency was calculated by dividing the net photosynthetic rate by the intercellular CO_2_ concentration. Photosystem II (PSII) activity, such as minimum fluorescence, maximum fluorescence, quantum yield of PSII, photochemical efficiency of PSII, photochemical quenching, non-photochemical quenching, and photosynthetic electron transport rate, was calculated according to the methods described by Maxwell and Johnson (2000).

### Effects on Biomass Productivity by Drought Conditions and Elevated CO_2_ Levels

Growth parameters, including plant height, number of leaves, number of branches, stem diameter, leaf thickness, leaf area, leaf fresh weight, dry weight, and root-shoot fresh and dry weights, were measured at the conclusion of the experiment on day 10. Plants were removed from pots, and the stem was cut from the roots. Root fresh weight was measured after careful washing and air drying. Stem, leaves, and roots were placed in a separate paper bag and dried in an oven at 70°C for 72 h.

### Statistical Analysis

Data for gas exchange parameters were presented in graphs along with the SE of each mean, and the graphs were prepared using the SigmaPlot version 11 software. To investigate the effect of time of observation (time), water conditions (water), and CO_2_ levels (CO_2_), physiological parameters were analyzed with Statistix version 8.1 using three-way ANOVA. All the main effects and two- and three-way interactions were examined using the Tukey’s honestly significant difference (HSD) test at a 5% probability level. Data of growth parameters were subjected to two-way ANOVA to assess the effect of CO_2_ concentrations and water regimes on the growth of *A. longifolia* ssp. *longifolia*. Five replications (two plants per pot) were used for each combination of treatments. The significance among the treatment means was separated by using the Tukey’s HSD at *p* ≤ 0.05.

## Results

### Effects on Photosynthetic Response by Drought Conditions and Elevated CO_2_ Levels

**FIGURE 1:**
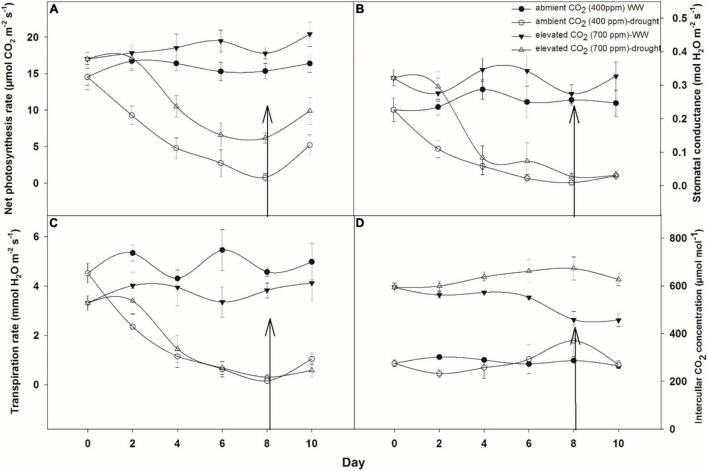
Effect of CO_2_ concentrations and moisture conditions on photosynthetic rate **(A)**, stomatal conductance **(B)**, transpiration rate **(C)**, and intercellular CO_2_ concentration **(D)** of *A. longifolia* ssp. *longifolia*. Nail on the data point represents the SE of the mean, and vertical arrow line after day 8 represents the addition of water in the drought treatments. WW, well-watered.

Photosynthetic activities were measured for *A. longifolia* ssp. *longifolia* when grown under two moisture regimes (i.e., well-watered and drought) at 400 ppm CO_2_ concentration (aCO_2_ concentration) and 700 ppm CO_2_ concentration (eCO_2_ concentration). Figure 1A shows that eCO_2_ concentration increased the net photosynthetic rate by 2–4 μmol CO_2_ m^–2^ s^–1^ when compared to that with aCO_2_ concentration under well-watered conditions. However, a variable response was noted with both CO_2_ concentrations when *A. longifolia* ssp. *longifolia* was grown under drought conditions. At aCO_2_ concentration, the net photosynthetic rate decreased to 0.07 μmol CO_2_ m^–2^ s^–1^ at day 8, whilst plants under eCO_2_ concentration recorded a net photosynthetic rate of 6.17 μmol CO_2_ m^–2^ s^–1^ on the same day. After all these measurements, water was added to the drought treatments on day 8 to investigate the recovery response of the plants in terms of the net photosynthetic rate. Photosynthesis was recovered at a high rate with eCO_2_ and increased from 6.17 to 9.8 μmol CO_2_ m^–2^ s^–1^, whilst under aCO_2_ concentration, it increased from 0.17 to 5.1 μmol CO_2_ m^–2^ s^–1^ (Figure 1A). The ANOVA results showed that time (*p* = 0.001), water (*p* < 0.001), CO_2_ (*p* < 0.001), time × water (*p* = 0.001), and time × CO_2_ (*p* = 0.001) were significant for photosynthetic rate of *A. longifolia*, whereas the interaction of water × CO_2_ and time × water × CO_2_ was non-significant (Table 1).

**TABLE 1:** Statistical significances for physiological parameters of *A. longifolia* ssp. *longifolia* in response to time of observation, water conditions, and CO_2_ levels.

Parameter	Time		Water		CO_2_		Time × water		Time × CO_2_		Water × CO_2_		Time × water × CO_2_	
	*p*-value	HSD at 0.05	*p*-value	HSD at 0.05	*p*-value	HSD at 0.05	*p*-value	HSD at 0.05	*p*-value	HSD at 0.05	*p*-value	HSD at 0.05	*p*-value	HSD at 0.05
Photosynthetic rate	0.001	3.82	0.000	1.51	0.000	1.50	0.000	6.22	0.001	6.22	0.466	2.80	0.158	9.83
Stomatal conductance	0.000	0.06	0.000	0.02	0.196	0.03	0.000	0.11	0.372	0.10	0.062	0.05	0.005	0.17
Transpiration rate	0.000	0.95	0.000	0.37	0.580	0.38	0.000	1.55	0.383	1.55	0.010	0.70	0.033	2.45
Intercellular CO_2_ concentration	0.000	74.6	0.000	29.41	0.000	29.41	0.000	121.55	0.000	121.55	0.000	54.80	0.000	192.15
Water use efficiency	0.050	23.19	0.791	9.14	0.041	9.13	0.045	37.76	0.123	37.75	0.107	17.03	0.231	59.70
Intrinsic water use efficiency	0.02	365.9	0.910	144.21	0.043	144.22	0.272	595.90	0.182	595.80	0.083	268.70	0.309	942.12
Instantaneous carboxylation efficiency	0.893	0.001	0.080	0.001	0.103	0.001	0.105	0.001	0.120	0.001	0.102	0.001	0.098	0.001
Photosynthetic electron transport rate	0.017	23.50	0.000	9.26	0.412	9.26	0.000	38.26	0.110	38.27	0.356	17.25	0.858	60.49
Minimum fluorescence	0.003	37.35	0.487	4.71	0.050	4.72	0.471	60.82	0.066	60.82	0.081	27.42	0.940	96.15
Maximum fluorescence	0.000	113.99	0.000	44.99	0.042	44.91	0.133	185.61	0.445	185.61	0.185	83.68	0.911	293.40
Quantum yield of PSII	0.016	0.55	0.000	0.02	0.040	0.02	0.000	0.09	0.011	0.09	0.359	0.04	0.852	0.14
Photochemical efficiency of PSII	0.000	0.05	0.000	0.02	0.050	0.02	0.024	0.08	0.621	0.09	0.160	0.03	0.916	0.13
Photochemical quenching	0.000	0.08	0.000	0.03	0.062	0.03	0.000	0.14	0.008	0.14	0.230	0.06	0.741	0.22
Non-photochemical quenching	0.000	0.24	0.000	0.09	0.310	0.09	0.037	0.39	0.618	0.39	0.880	0.17	0.674	0.62

*A. longifolia* ssp. *longifolia* exhibited a significant difference in stomatal conductance under eCO_2_ and aCO_2_ concentrations (Figure 1B). eCO_2_ inhibited the stomatal conductance compared to that with aCO_2_ under well-watered conditions. Drought stress resulted in a progressive decline in stomatal conductance up to day 8 with aCO_2_ (0.009 mol H_2_O m^–2^ s^–1^). However, this decline was less with eCO_2_, since 0.027 mol H_2_O m^–2^ s^–1^ stomatal conductance was recorded on day 8. After the addition of water to drought treatments on day 8, stomatal conductance recovered slightly, but this recovery was larger with eCO_2_ than that with aCO_2_. The results of ANOVA in Table 1 indicate that the stomatal conductance *of A. longifolia* spp. *longifolia* in response to time, water, CO_2_, and time × water was significant (*p* < 0.04).

The eCO_2_ concentration inhibited the transpiration rate under well-watered conditions compared to that with aCO_2_ (Figure 1C). The transpiration rate reduced progressively with an increase in the duration of drought under both CO_2_ regimes. During the initial days of drought stress, transpiration did not reduce significantly with eCO_2_, whilst aCO_2_ reduced the transpiration rate linearly, and it was measured at 0.6 mmol H_2_O m^–2^ s^–1^ on the 8th day of observation. The addition of water to drought treatments after the 8th day of observation resulted in higher recovery (from 0.6 to 1.0 4 mmol H_2_O m^–2^ s^–1^) in the transpiration rate of *A. longifolia* ssp. *longifolia* with aCO_2_, compared to that when grown under eCO_2_. Response of transpiration rate of *A. longifolia* spp. *longifolia* to time, water, time × water, and water × CO_2_ was significant (Table 1).

When *A. longifolia* ssp. *longifolia* was grown under well-watered conditions, eCO_2_ caused an increase in intracellular CO_2_ concentration, being recorded between 450 and 590 μmol mol^–1^ compared with aCO_2_ where intercellular CO_2_ concentration was between 270 and 300 μmol mol^–1^ (Figure 1D). Drought stress exerted significant effects on intercellular CO_2_ concentration, and it increased with an increase in drought duration under both CO_2_ concentrations, attaining maximum value on the 8th day of measurement. An increase in intracellular CO_2_ concentration was higher with eCO_2_ compared to that with aCO_2_. The addition of water to drought treatments on the 8th day slightly reduced the value of intercellular CO_2_ concentration, whilst it was significantly reduced under aCO_2_ with the addition of water. The ANOVA results showed that the main effects (time, water, and CO_2_) and two-way or three-way interactions for intracellular CO_2_ concentration were significant (*p* < 0.02).

**FIGURE 2:**
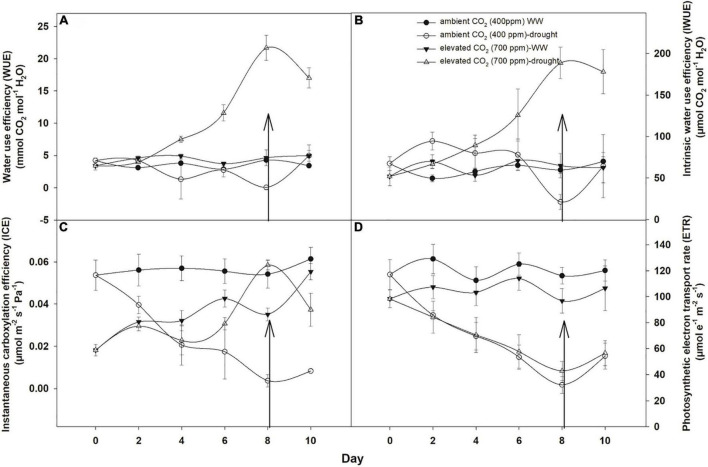
Effect of CO_2_ concentrations and moisture conditions on water use efficiency **(A)**, intrinsic water use efficiency **(B)**, instantaneous carboxylation efficiency **(C)**, and photosynthetic electron transport rate **(D)** of *A. longifolia* ssp. *longifolia*. Nail on the data point represents the SE of the mean, and vertical arrow line after day 8 represents the addition of water in the drought treatments. WW, well-watered.

Under well-watered conditions, eCO_2_ increased the water use efficiency by 0.76 mmol CO_2_ mol^–1^ H_2_O in comparison with that with aCO_2_, whereas the effects of drought on water use efficiency of *A. longifolia* ssp. *longifolia* were different under both CO_2_ concentrations. eCO_2_ linearly increased the water use efficiency and recorded a maximum on the 8th day of measurement (21.6 mmol CO_2_ mol^–1^ H_2_O). Whilst drought conditions reduced the water use efficiency of *A. longifolia* ssp. *longifolia* when grown under aCO_2_, the recorded minimum water use efficiency (0.038 mmol CO_2_ mol^–1^ H_2_O) was measured on the 8th day. The addition of water to drought treatments when grown under aCO_2_ recovered the water use efficiency by 4.09 mmol CO_2_ mol^–1^ H_2_O till the 10th day of measurement. In contrast to aCO_2_, the recovery in water use efficiency did not occur with eCO_2_, and it was reduced with the addition of water to drought treatments when grown under eCO_2_ concentration (Figure 2A). The ANOVA results showed that the effect of time, CO_2_, and time × water on water use efficiency of *A. longifolia* spp. *longifolia* was significant (Table 1).

Intrinsic water use efficiency of *A. longifolia* ssp. *longifolia* was the same under both CO_2_ concentrations when grown under well-watered conditions (Figure 2B). However, the effect of drought was different for intrinsic water use efficiency under both CO_2_ concentrations. eCO_2_ in drought conditions increased the intrinsic water use efficiency linearly up to the 8th day of measurement. Whilst aCO_2_ sustained the intrinsic water use efficiency under drought conditions till the 6th day of measurement, after this time, the intrinsic water use efficiency decreased to a minimum value (21.1 μmol CO_2_ mol^–1^ H_2_O). The addition of water to the drought treatments had no effects in terms of recovery of intrinsic water use efficiency of *A. longifolia* ssp. *longifolia* when grown under eCO_2_. In contrast, significant recovery in intrinsic water use efficiency was noted for aCO_2_ with the addition of water on the 8th day of measurement (Figure 2B). The ANOVA showed that the effect of time (*p* = 0.02) and CO_2_ (*p* = 0.04) on intrinsic water use efficiency was significant.

Instantaneous carboxylation efficiency of *A. longifolia* ssp. *longifolia* was higher with aCO_2_ than that with eCO_2_ when grown under well-watered conditions (Figure 2C). Drought stress had variable effects on instantaneous carboxylation efficiency under both CO_2_ concentrations. eCO_2_ under drought conditions had no effects on instantaneous carboxylation efficiency up to the 4th day of observation. Subsequently, it increased the instantaneous carboxylation efficiency and recorded the maximum value (0.05 μmol m^–2^ s^–1^ Pa^–1^) on the 8th day of measurement. This contrasts with aCO_2_ under drought conditions, which progressively decreased the instantaneous carboxylation efficiency with an increase in drought duration. The addition of water to drought treatments when grown under eCO_2_ decreased the instantaneous carboxylation efficiency, while the addition of water to aCO_2_ treatment did not affect instantaneous carboxylation efficiency (Figure 2C). According to the results of ANOVA, the effect of time, water, and CO_2_ on instantaneous carboxylation efficiency was non-significant.

Under well-watered conditions, the photosynthetic electron transport rate was higher with aCO_2_ concentration than that with eCO_2_ concentration (Figure 2D). Drought significantly affected the photosynthetic electron transport efficiency with every increment in the drought days, and the photosynthetic electron transport rate reached a minimum value (32 μmol e^–1^ m^–2^ s^–1^ for aCO_2_ and 42 μmol e^–1^ m^–2^ s^–1^ for eCO_2_) on the 8th day of measurement under both CO_2_ concentrations. The addition of water to drought treatments recovered the photosynthetic electron transport rate under both CO_2_ concentrations. The photosynthetic electron transport rate of *A. longifolia* spp. *longifolia* was significant in response to time and CO_2_; however, all the interactions were non-significant (Table 1).

**FIGURE 3:**
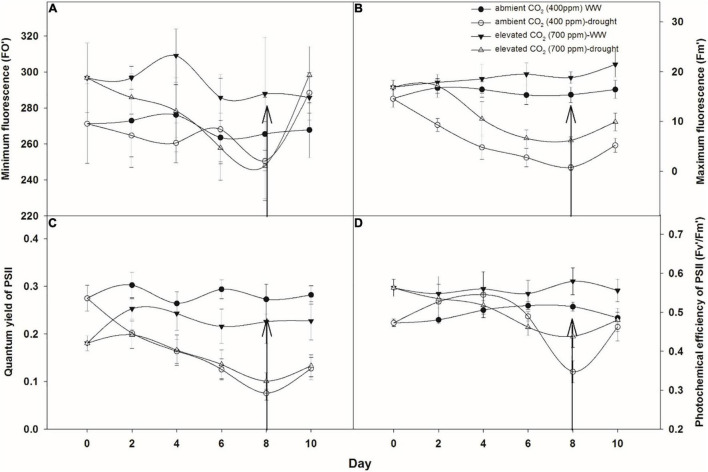
Effect of CO_2_ concentrations and moisture conditions on minimum fluorescence **(A)**, maximum fluorescence **(B)**, quantum yield of photosystem II (PSII) **(C)**, and photochemical efficiency of PSII **(D)** of *A. longifolia* ssp. *longifolia*. Nail on the data point represents the SE of the mean, and vertical arrow line after day 8 represents the addition of water in the drought treatments. WW, well-watered.

The minimum and maximum fluorescence values of *A. longifolia* ssp. *longifolia* when grown under well-watered conditions were higher with eCO_2_ compared to those with aCO_2_ (Figures 3A,B). The effect of drought was more pronounced on minimum fluorescence under eCO_2_ as fluorescence decreased linearly till the 8th day of observation with an increase in drought duration under eCO_2_. In comparison, aCO_2_ under drought conditions had no effects on minimum fluorescence till the 6th day of observation, and after that, it began to reduce on day 8 (Figure 3A). The addition of water to drought treatments recovered the minimum fluorescence for both CO_2_ concentrations. Maximum fluorescence under drought conditions was higher with eCO_2_ when compared to that with aCO_2_, and a decline in maximum fluorescence occurred in both CO_2_ concentrations with an increase in drought duration till the 8th day of measurement. The addition of water to drought treatments slightly recovered the maximum fluorescence in both CO_2_ concentrations (Figure 2D). The effect of time (*p* = 0.003) and CO_2_ (*p* = 0.05) on minimum fluorescence was significant, whereas the effect of time (*p* < 0.001), water (*p* < 0.001), and CO_2_ (*p* = 0.042) on maximum fluorescence was significant (Table 1).

The quantum yield of PSII was relatively higher when *A. longifolia* ssp. *longifolia* was grown under aCO_2_ in well-watered conditions (Figure 3C). Drought stress decreased the yield progressively in both CO_2_ concentrations. eCO_2_ slightly mitigated the adverse effects of drought on the quantum yield of PSII as the yield was 0.1 on the 8th day of measurement compared to that with aCO_2_ where it was 0.07 on the same day (Figure 3C). The addition of water to drought treatments recovered the yield in the same way for both CO_2_ concentrations. The ANOVA table showed that time (*p* = 0.016), water (*p* < 0.001), CO_2_ (*p* = 0.040), time × CO_2_ (*p* < 0.001), and time × water (*p* = 0.011) have significant effects on quantum yield of PSII (Table 1). Under well-watered conditions, the photochemical efficiency of PSII was significantly higher with eCO_2_ compared to that with aCO_2_ throughout the observation period (days 0–10) (Figure 3D). Data showed that eCO_2_ mitigated the adverse effects of drought on the photochemical efficiency of PSII as no decline in photochemical efficiency of PSII occurred until day 4, and after that, the photochemical efficiency of PSII slightly reduced till the 8th day of observation. The drought had drastic effects on the photochemical efficiency of PSII under aCO_2_ which decreased to 0.34. However, the addition of water to drought treatments when grown under aCO_2_ recovered the photochemical efficiency of PSII significantly compared to that in eCO_2_ (Figure 3D). The ANOVA table showed that time (*p* < 0.001), water (*p* < 0.001), CO_2_ (*p* = 0.050), and time × CO_2_ (*p* = 0.024) have significant effects on the photochemical efficiency of PSII (Table 1).

**FIGURE 4:**
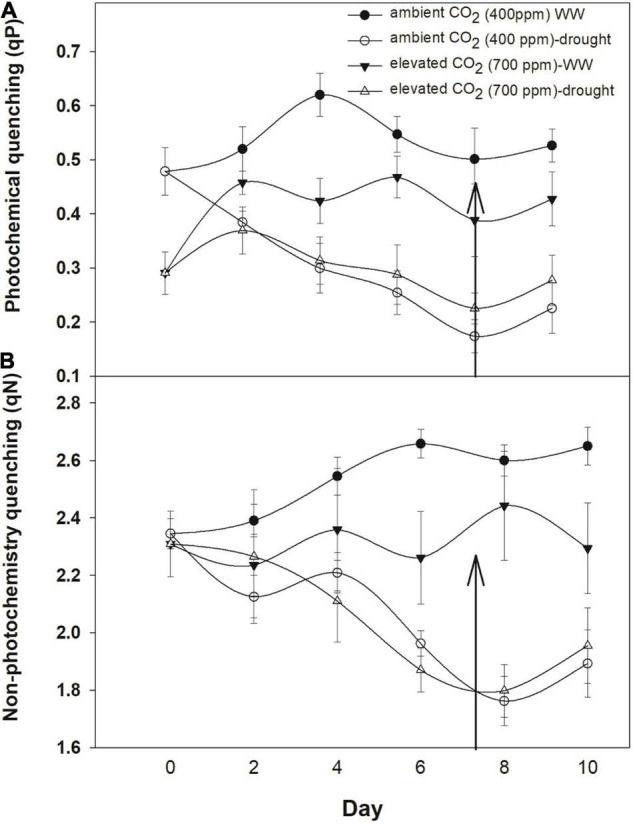
Effect of CO_2_ concentrations and moisture conditions on photochemical quenching **(A)** and non-photochemical quenching **(B)** of *A. longifolia* ssp. *longifolia*. Nail on the data point represents the SE of the mean, and vertical arrow line after day 8 represents the addition of water in the drought treatments. WW, well-watered.

Photochemical and non-photochemical quenching was higher with aCO_2_ when *A. longifolia* ssp. *longifolia* was grown under well-watered conditions (Figures 4A,B). eCO_2_ mitigated the adverse effects of drought on photochemical quenching, and a decline in this parameter was less with an increase in drought duration as compared to that with aCO_2_. eCO_2_ had no effects on non-photochemical quenching under drought conditions. The addition of water to drought treatments recovered the photochemical and non-photochemical quenching under both CO_2_ concentrations (Figures 4A,B). The results of ANOVA indicated that the effect of time, water, and time × water was significant on photochemical and non-photochemical quenching of *A. longifolia* (Table 1).

### Effects on Biomass Productivity by Drought Conditions and Elevated CO_2_ Levels

**FIGURE 5:**
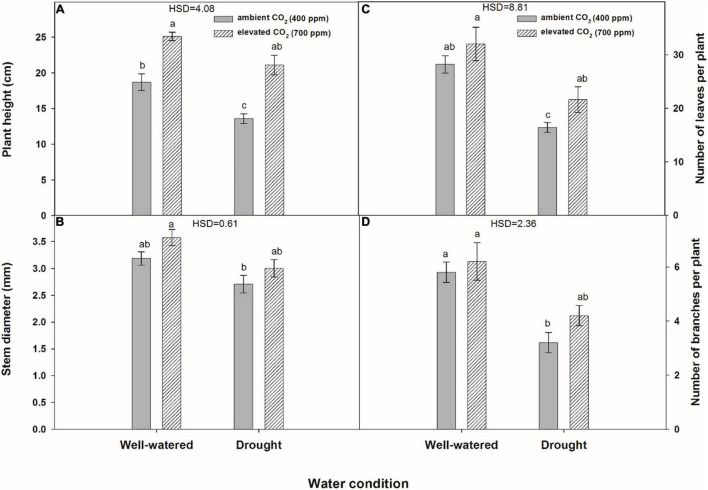
Effect of CO_2_ concentrations and moisture conditions on plant height **(A)**, stem diameter **(B)**, number of leaves per plant **(C)**, and number of branches per plant **(D)** of *A. longifolia* ssp. *longifolia*. Nail on the bar represents the SE of the mean.

**FIGURE 6:**
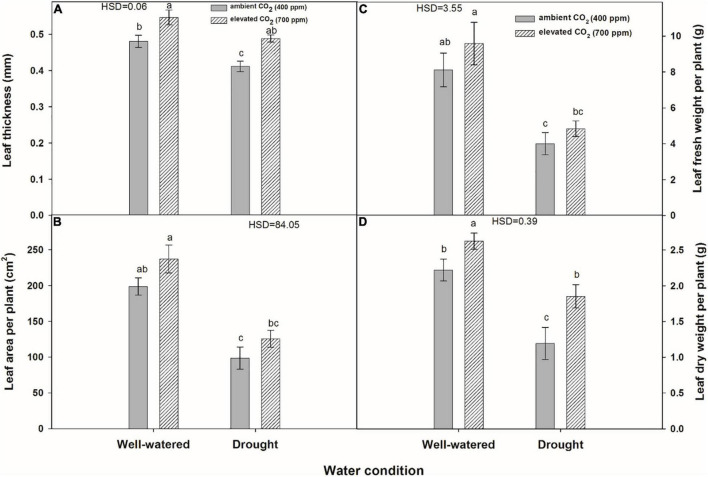
Effect of CO_2_ concentrations and moisture conditions on leaf thickness **(A)**, leaf area per plant **(B)**, leaf fresh weight per plant **(C)**, and leaf dry weight per plant **(D)** of *A. longifolia* ssp. *longifolia*. Nail on the bar represents the SE of the mean.

Plant height was significantly affected by water regimes and CO_2_ concentrations (Figure 5A). eCO_2_ produced 6.4 cm taller plants (25.1 cm) of *A. longifolia* ssp. *longifolia* under well-watered conditions compared with aCO_2_ (18.7 cm). Under drought conditions, eCO_2_ mitigated the adverse effects of drought on the height of *A. longifolia* ssp. *longifolia* and recorded 21.1 cm taller plants. Plants subjected to drought when grown under aCO_2_ attained a height of only 13.6 cm. Under well-watered conditions, the number of leaves per plant was statistically the same with both CO_2_ concentrations. Stem diameter was not improved with eCO_2_ under well-watered conditions in comparison with aCO_2_, whilst eCO_2_ enhanced the stem diameter by 0.3 mm when grown under drought conditions (Figure 5B). Drought significantly affected the number of leaves per plant under both CO_2_ concentrations, but eCO_2_ slightly improved the number of leaves by mitigating the drought effects, hence, producing four more leaves than that with aCO_2_ (Figure 5C). The number of branches was the same for both CO_2_ concentrations under well-watered conditions (Figure 5D). However, eCO_2_ mitigated the adverse effects of drought and produced a higher number of branches per plant (4.2) compared to that with aCO_2_ where branches (3.2) were produced under drought conditions. Drought stress affected the leaf thickness and leaf area per plant under both CO_2_ concentrations, and this effect was more when plants were grown under aCO_2_ (Figures 6A,B). The effect of eCO_2_ was significant for leaf thickness and non-significant for leaf area per plant when *A. longifolia* ssp. *longifolia* was grown under well-watered conditions. eCO_2_ increased the plant height, stem diameter, number of leaves, and number of branches by 34, 12, 13, and 6%, respectively, over aCO_2_ under well-watered conditions. In contrast, under drought conditions, an increase of 55, 10, 31, and 31% in plant height, stem diameter, number of leaves, and number of branches, respectively, was observed with eCO_2_ over aCO_2_ (data not shown).

**FIGURE 7:**
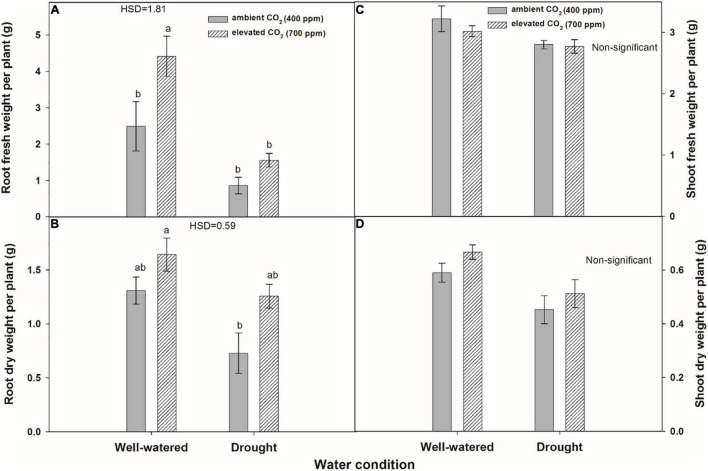
Effect of CO_2_ concentrations and moisture conditions on root fresh weight per plant **(A)**, root dry weight per plant **(B)**, shoot fresh weight per plant **(C)**, and shoot dry weight per plant **(D)** of *A. longifolia* ssp. *longifolia*. Nail on the bar represents the SE of the mean.

Leaf fresh weight was significantly affected by water regimes, and drought caused the lowest fresh weight of leaves per plant (4.8 g) under aCO_2_ (Figure 6C). Leaf dry weight was significantly affected by water regimes and CO_2_ concentrations. eCO_2_ produced higher leaf dry weight under both water conditions when compared to that with aCO_2_ (Figure 6D). The effect of water regimes and CO_2_ concentrations was significant for root fresh weight per plant of *A. longifolia* ssp. *longifolia* (Figure 7A). eCO_2_ recorded maximum fresh weight of root when grown under well-watered conditions. However, eCO_2_ did not enhance the root fresh weight under drought conditions in comparison with that of aCO_2_. Root dry weight was also enhanced by eCO_2_ when *A. longifolia* ssp. *longifolia* was grown under well-watered conditions (Figure 7B). The effect of water conditions and CO_2_ concentrations was non-significant for the shoot fresh and shoot dry weights of *A. longifolia* ssp. *longifolia* (Figures 7C,D). eCO_2_ increased the leaf fresh weight, leaf dry weight, root fresh weight, and root dry weight by 18, 19, 77, and 25% under well-watered conditions and 8, 57, 39, and 38% under drought conditions, respectively, over aCO_2_ (data not shown).

## Discussion

eCO_2_ and drought stress are the main components of climate change that directly affect the physiology and morphology of plants (Lobell and Gourdji, 2012; Salazar-Parra et al., 2012; Chadha et al., 2020; Dawood et al., 2021). This study exhibits that CO_2_ concentrations (ambient at 400 ppm and elevated at 700 ppm) had variable effects on gas exchange, water use efficiency, PSII activities, and growth of *A. longifolia* ssp. *longifolia* when grown under well-watered and drought conditions. Drought conditions had negative effects on photosynthetic activity, water use efficiency, and growth of *A. longifolia* ssp. *longifolia*, but eCO_2_ mitigated the adverse effects of drought on the physiological and morphological parameters of this plant. Prior study showed that drought stress causes various responses of photosynthetic mechanisms depending upon species (Prieto et al., 2009; Weller et al., 2020). Furthermore, leaf photosynthesis is generally believed to increase with eCO_2_ even under stressful environments (Meibaum et al., 2012). In our study, the net photosynthetic rate of *A. longifolia* ssp. *longifolia* leaves increased by 12% with eCO_2_. According to Evans et al.’s (2000) study on growth and gas exchange, measurements of nine *Acacia* species were recorded to be enhanced under 700 μl L^–1^ CO_2_ concentration. A study by Ibrahim et al. (2020) reported that *Acacia mangium* was well adapted to higher temperatures and eCO_2_ compared to native health forest species, including *Buchanania arborescens* Blume, *Dillenia suffruticosa* Martelli, *Calophyllum inophyllum* L., and *Ploiarium alternifolium* Melch. eCO_2_ induced a low transpiration rate under well-watered and drought conditions. The decline in transpiration rate might have been due to the stomatal closure as a result of the effect of eCO_2_ and drought. It is postulated that eCO_2_ increases cytosolic-free calcium within guard cells, which may act as a messenger in the signal transduction pathway for maintaining the turgor pressure of the guard cells (Kim et al., 2010). Low transpiration rate and stomatal conductance of *A. longifolia* ssp. *longifolia* under eCO_2_ conserve soil moisture (Habermann et al., 2019), and this increases the net photosynthetic rate and growth under eCO_2_. Soil moisture conservation in other species exposed to eCO_2_ has been observed (Wullschleger et al., 2002; Qaderi et al., 2013; Miranda-Apodaca et al., 2018; Dikšaitytė et al., 2019). According to Yu et al. (2012), plants grown under eCO_2_ in drought conditions resulted in greater stomatal closure than that of the plants grown under aCO_2_. A study by Weller et al. (2021) showed that stomatal conductance of *Amaranthus retroflexus* L. (redroot pigweed) was reduced under eCO_2_. Furthermore, eCO_2_ reduced the drought-induced damage, and plant recovery was notable upon re-watering the plants grown under water stress. However, in previous studies, it was noted that the degree of recovery depends upon species and duration of drought, as barley plants were not able to recover stomatal closure when plants were re-watered after 16 days of drought stress under eCO_2_ (Robredo et al., 2007). In our study, stomatal conductance of *A. longifolia* ssp. *longifolia* was recovered with the addition of water to drought-treated plants. In contrast, higher transpiration rate and stomatal conductance of *A. longifolia* ssp. *longifolia* under aCO_2_ with both moisture conditions caused a faster decrease in soil moisture contents which affected the photosynthesis activity and growth of the plant. These findings share some similarities with tall fescue, in which a lower stomatal conductance was observed under drought conditions in the aCO_2_ treatment (Yu et al., 2012). Furthermore, our findings are inconsistent with those of Robredo et al. (2007) who concluded that under eCO_2_, water stress was developed more slowly due to a slower rate of moisture depletion. Intercellular CO_2_ concentration was increased with an increase in drought duration, and this showed higher increase with eCO_2_, which could have been due to the combined effect of eCO_2_ and drought, thereby causing stomatal closure. Conversely, our findings are in line with those of Robredo et al. (2007) and Wang et al. (2015) who reported that eCO_2_ caused stomatal closure with high intercellular CO_2_ concentration at 700 ppm, which led to a higher rate of carbon assimilation.

Under well-watered conditions, water use efficiency of *A. longifolia* ssp. *longifolia* increased by 0.75 mmol CO_2_ mol^–1^ H_2_O under eCO_2_ in comparison with that of aCO_2_. Water use efficiency was seen to be further enhanced when plants were grown under drought conditions. This might have been the result of decreased transpiration rate under eCO_2_. Previous studies have shown that eCO_2_ increased water use efficiency by lowering the transpiration rate and moisture depletion at eCO_2_ (Schmid et al., 2016; Tom-Dery et al., 2019; Hebbar et al., 2020; Ma et al., 2020). For example, photosynthetic activities and water use efficiency of invasive *A. mangium* were reported to be stimulated by eCO_2_ when compared to that with native *B. arborescens* and *D. suffruticosa* (Ibrahim et al., 2020). Therefore, regarding the increase in water use efficiency of *A. longifolia* ssp. *longifolia* under eCO_2_, our results are supported by Saxe et al. (1998) and Gilbert et al. (2011) who reported that intrinsic water use efficiency increased by 25–30% as a result of reduced stomatal conductance and enhanced photosynthetic rate under eCO_2_. Soil moisture conservation due to eCO_2_ concentration has been observed in other species (Wullschleger et al., 2002; Madhu and Hatfield, 2013; Kimball, 2016), and this may reduce the impact of limited water conditions on growth (Reddy et al., 2010). Our results are in accordance with the findings of Pérez-López et al. (2013) and Wang et al. (2015) who reported that instantaneous carboxylation efficiency declined with eCO_2_.

Drought significantly affected the photosynthetic electron transport rate under both CO_2_ concentrations, but this decline was less marked with eCO_2_. A higher rate of photosynthetic electron transport occurring in a drought stress environment coupled with eCO_2_ was also reported by Kitao et al. (2007). Drought stress decreased the quantum yield of PSII, whilst eCO_2_ mitigated the adverse effects of drought on the quantum yield of PSII in our study. Similarly, the photochemical efficiency of PSII was also increased with eCO_2_, which led to the conclusion that a CO_2_-enriched environment reduces the risk of damage by drought stress. These findings are also supported by Perveen et al. (2010), Zhao et al. (2010), and Wang et al. (2018) who showed that eCO_2_ decreased the quantum yield of PSII and increased the photochemical efficiency of PSII. Drought stress caused a decline in photochemical quenching; however, eCO_2_ reduced the rate of decline even with an increase in drought duration. This suggested that eCO_2_ reduced the risk of photoinhibition (Arena et al., 2011).

Water stress generally has negative effects on plant growth, but under eCO_2_, growth appears to have a positive effect (Serret et al., 2018). The interactive effect of drought and eCO_2_ is important to consider when predicting the effects of climate change on plant species (Martínez-Carrasco et al., 2005; Battaglia and Bruce, 2017). Our results showed that eCO_2_ significantly increased plant height, number of leaves, stem diameter, leaf thickness, leaf area, leaf dry weight, and root dry weight of *A. longifolia* ssp. *longifolia* when compared to aCO_2_ under both moisture conditions. This enhanced growth with eCO_2_ might have been due to increased photosynthesis and water use efficiency, and reduced stomatal conductance resulting in decreased transpiration rate. Ziska et al. (2019) predicted that plant biomass would increase as a result of increased photosynthetic rate and water use efficiency. The reports of Qaderi et al. (2006) showed that eCO_2_ increased the availability of building blocks for the production of proteins and structural molecules, which increased the leaf area and specific leaf mass. This was supported by the observation that plants grown under aCO_2_ and water stress had the lowest biomass productions. Previous studies have shown that eCO_2_ alleviated the drought impacts by reinforcement in CO_2_ uptake, decreasing the oxidative pressure in the chloroplast electron transport chain, changing the biomass accumulation, and altering the partitioning to favor water acquisition. All these changes enable the plants to cope more effectively with water shortages (Avila et al., 2020). Another study demonstrated that drought-imposed limitations on photosynthesis were based on a significant decrease in stomatal conductance and intercellular concentration (Salmon et al., 2020). Given that the increased photosynthesis under eCO_2_ resulted in more ATP use for ribulose 1,5-bishosphate (RuBP) regeneration (López-Calcagno et al., 2020). eCO_2_ levels also improve the allometric adjustment linked to drought tolerance (Avila et al., 2020). The aggressive nature of *A. longifolia* ssp. *longifolia* for growth under eCO_2_ will affect the growth and wellbeing of native species by altering the ecosystem and introducing direct competition for resources in a new environment.

## Conclusion

This study reveals that eCO_2_ improved the photosynthetic activity, water use efficiency, and general growth parameters of *A. longifolia* ssp. *longifolia* under both well-watered and drought conditions. Therefore, this species is able to compensate for a lack of soil moisture with greater growth of its root and shoot, and it takes an advantage of eCO_2_ to increase overall biomass. This ability to grow under limited water availability with eCO_2_ indicated that this species will be able to adapt to new environments even under severe variation in climatic conditions, and foreshadows its likelihood of invasion into new areas. This species has a potential threat to the ecosystem in future due to changing climatic conditions.

## Data Availability Statement

The raw data supporting the conclusions of this article will be made available by the authors, without undue reservation.

## Author Contributions

SKF conceived and designed the study. MMJ conducted the experiments. All authors were involved in writing, reviewing the manuscript.

## Conflict of Interest

The authors declare that the research was conducted in the absence of any commercial or financial relationships that could be construed as a potential conflict of interest.

## Publisher’s Note

All claims expressed in this article are solely those of the authors and do not necessarily represent those of their affiliated organizations, or those of the publisher, the editors and the reviewers. Any product that may be evaluated in this article, or claim that may be made by its manufacturer, is not guaranteed or endorsed by the publisher.

## Abbreviations

aCO_2_: ambient CO_2_
eCO_2_: elevated CO_2_
PPFD: photosynthetic photon flux density
PSII: photosystem II
RuBP: ribulose 1,5-bishosphate.
